# Immobilization of α-Amylase from *Anoxybacillus* sp. SK3-4 on ReliZyme and Immobead Supports

**DOI:** 10.3390/molecules21091196

**Published:** 2016-09-09

**Authors:** Ummirul Mukminin Kahar, Mohd Helmi Sani, Kok-Gan Chan, Kian Mau Goh

**Affiliations:** 1 Faculty of Biosciences and Medical Engineering, Universiti Teknologi Malaysia, Skudai 81310, Johor, Malaysia; ummirulmukminin@gmail.com (U.M.K.); helmisani@fbb.utm.my (M.H.S.); 2 Division of Genetics and Molecular Biology, Institute of Biological Sciences, Faculty of Science, University of Malaya, Kuala Lumpur 50603, Malaysia; kokgan@um.edu.my

**Keywords:** *Anoxybacillus*, amylase, glycoside hydrolase, immobilize, Immobead, ReliZyme, Sepabeads, starch, thermophiles, thermostable enzyme

## Abstract

α-Amylase from *Anoxybacillus* sp. SK3-4 (ASKA) is a thermostable enzyme that produces a high level of maltose from starches. A truncated ASKA (TASKA) variant with improved expression and purification efficiency was characterized in an earlier study. In this work, TASKA was purified and immobilized through covalent attachment on three epoxide (ReliZyme EP403/M, Immobead IB-150P, and Immobead IB-150A) and an amino-epoxide (ReliZyme HFA403/M) activated supports. Several parameters affecting immobilization were analyzed, including the pH, temperature, and quantity (mg) of enzyme added per gram of support. The influence of the carrier surface properties, pore sizes, and lengths of spacer arms (functional groups) on biocatalyst performances were studied. Free and immobilized TASKAs were stable at pH 6.0–9.0 and active at pH 8.0. The enzyme showed optimal activity and considerable stability at 60 °C. Immobilized TASKA retained 50% of its initial activity after 5–12 cycles of reuse. Upon degradation of starches and amylose, only immobilized TASKA on ReliZyme HFA403/M has comparable hydrolytic ability with the free enzyme. To the best of our knowledge, this is the first report of an immobilization study of an α-amylase from *Anoxybacillus* spp. and the first report of α-amylase immobilization using ReliZyme and Immobeads as supports.

## 1. Introduction

*Anoxybacillus* spp. are thermophilic bacteria that grow optimally at temperatures ranging from 50 to 65 °C [1,2,3,4]. *Anoxybacillus* sp. SK3-4 (= DSM 28779 = KCTC 33552 = MCC 2593, 16S rRNA GenBank accession number: GQ184213) was isolated from a hot spring in Malaysia [5,6]. The sequenced genome of strain SK3-4 (NCBI Bioproject accession number: PRJNA174378) revealed several genes encoding glycoside hydrolases (GHs) known to degrade starch [7], and some of these enzymes have been extensively studied by our research group [8,9,10,11,12,13,14]. α-Amylases (EC 3.2.1.1) hydrolyze α-1,4-glycosidic bonds of polysaccharides [15]. α-Amylase from *Anoxybacillus* sp. SK3-4 (ASKA) is a novel thermostable enzyme that produces a high level of maltose upon reaction with starch, which is useful for starch saccharification [2,8,9,10,15,16]. Janecek et al. [17] proposed that ASKA may be classified as a novel subfamily of glycoside hydrolase 13 (GH13). Recently, we constructed a truncated ASKA (TASKA) enzyme variant by removing the signal peptide and transmembrane region of ASKA [8]. Preliminary data on TASKA showed that the truncation increased the expression and purification efficiency of the recombinant enzyme [8]. The X-ray structure of TASKA indicated that the structure consists of three domains (i.e., domains A, B, and C) and four Ca^2+^ ion-binding sites [8].

The use of α-amylase in soluble form (free enzyme) in industrial starch-saccharification processes is often hampered by low enzyme stability and high production cost [15,16]. Free α-amylases often lose stability as a result of changes in viscosity, pH, temperature, friction, and osmotic pressure imposed during starch saccharification [15]. This limitation leads to the use of high-concentration α-amylase, which is not economically viable. Various strategies have been applied to improve α-amylase performance [2,15,16,17], including discovery of thermostable enzymes and protein modifications that have resulted in several commercial products [18,19]. Immobilization is another possible way to improve current starch-processing methods [16]. Immobilization of the enzyme may provide five main advantages: (i) efficient recovery and reusability of the enzyme; (ii) facile separation of the enzyme from the reaction mixture; (iii) simplicity of the overall design and performance control of bioreactors; (iv) increased enzyme activity; or (v) improvement of other catalytic features such as stability and specificity (selectivity) [20,21,22,23,24,25,26,27,28,29,30,31,32].

The methods for enzyme immobilization are divided into three main categories which are entrapment (encapsulation), cross-linking, and binding to a carrier [21,26,27]. Among these strategies, covalent attachment offers the advantage of forming stronger and more stable linkages between the enzyme and the support [20,21,33]. Examples of covalent attachment include epoxide and heterofunctional amino-epoxide-activated supports, and enzymes such as galactosidase, fructosyltransferase, acylase, phytase, protease, and lipase have been shown to effectively create better biocatalysts [34,35,36,37,38,39,40,41]. Supports that utilize epoxide and amino-epoxide groups exhibit short and long spacer arms, respectively [33], and these functional groups are highly stable over a long reaction period and wide pH range [20,33,38,42]. Epoxide and amino-epoxide groups allow multiple covalent attachment of enzymes to the support, and thus provide favorable structural rigidification that increases enzyme stability [24,43]. The amino acids for which effective binding occurs have nucleophilic groups (i.e., Lys, Cys, His, and Tyr) or, in a lesser preferable configuration, carboxylic amino acids present on the enzyme surface [44]. In some enzyme-support systems, some proteins are less reactive to the functional groups. To overcome this issue, two-step immobilization mechanisms have been developed that involve (i) rapid and mild enzyme adsorption to the support surface and (ii) promotion of covalent attachment between the adsorbed enzyme and the functional groups of the support [24].

The advantages of enzyme immobilization have been discussed extensively in many previous reports [24,26,27,30,43,45]. For instance, the immobilized enzymes are able to overcome inhibition of by undesired additives or high substrate/product concentrations that are not amenable to free enzymes. One possible reason for the improved properties is the structural distortion of binding sites or pockets upon enzyme binding on suitable supports [24,43,46]. In certain enzymes, immobilization increased the structural rigidification of the protein through multipoint attachment and thus prevented undesired effects caused by harsh reaction conditions or reagents [28,47]. In the case of multimeric enzymes, multisubunit attachment increased structural rigidification by maintaining protein conformation, ionic interactions, and intra- or inter-molecular interactions between subunits, hence avoiding negative impacts on enzyme activity and stability [31,43]. The rigidification may also lead to some minor distortion of the enzyme’s catalytic site, affecting the enzyme specificity [24,25]. It has also been demonstrated that immobilization could be combined with protein purification for simplification and improvement of enzyme stability, all in a one-step process [47].

Despite of collective advantages of immobilized enzymes, few industries have adopted this practice and applied it on a large scale. Examples of enzymes that have been immobilized for biotechnology applications include glucose isomerase, lipase, lactase, and penicillin G acylase [48]. Enzyme immobilization is hardly applied on an industrial scale when bulky or complex substrates are involved, probably because of low diffusional constraints [48,49], and other reasons suggested in earlier work [24,43,47,50]. However, researchers remain interested in the discovery of suitable supports for such applications. Additionally, DiCosimo et al. [48] highlighted that the price of enzymes is relatively low compared to the price of the whole reaction process; therefore immobilization of enzymes may appear to be unnecessarily tedious from the point of view of bioprocess engineers.

Many commercial, ready-to-use epoxide or amino-epoxide-activated supports are commercially available at an affordable price. Examples include Sepabeads, ReliZyme (a derivative of Sepabeads), Immobead, and Purolite. These acrylic-based macroporous supports also offer high mechanical stability, high resistance towards microbial contamination, and low swelling in water, which makes them suitable for various laboratory or industrial applications [20,21,22,51]. Furthermore, the material used in the production of such supports is safe for use in food processing [52]. Here, we aimed to optimize covalent TASKA immobilization on a solid support. In this study, we employed 4 commercial epoxide and amino-epoxide supports, including ReliZyme EP403/M, ReliZyme HFA403/M, Immobead IB-150P, and Immobead IB-150A. The matrix materials, surface properties, functional groups and densities, particle sizes and surface areas, and pore information for each carrier are summarized in Table 1. We first optimized the immobilization conditions by using different pH, temperatures, and enzyme quantities (mg) added per g of support material. The immobilization performance of each support was then determined in terms of the protein-loading efficiency, activity retention, and activity recovery. Based on experimental data, attempts were made to correlate physical properties of each carrier (i.e., surface properties, pore sizes, and functional groups) with performance of the immobilized enzymes. Subsequently, the immobilized enzymes were characterized and compared with the free enzyme. This study shows that the highest enzyme activity retention and activity recovery was achieved using ReliZyme HFA403/M. We propose that this may be owing to multiple physical properties of the support (not only the pore diameter), such as its hydrophilic surface, pore size, and extended length of its functional groups.

## 2. Results

### 2.1. Purification of TASKA

**Figure 1:**
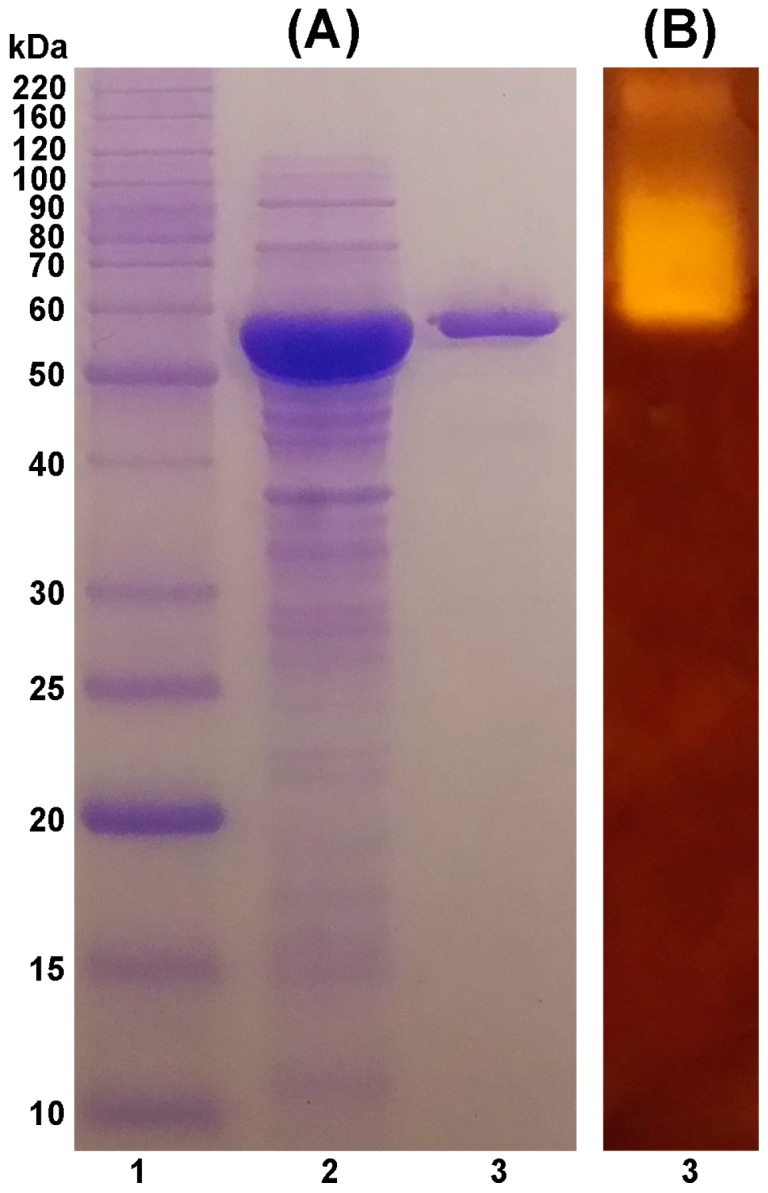
(**A**) SDS-PAGE (12%) separation of TASKA; (**B**) Zymogram for the amylolytic activity of TASKA. Lane 1, Molecular mass protein marker (BenchMark™ Protein Ladder, Life Technologies, Carlsbad, CA, USA); Lane 2, crude enzyme; Lane 3, purified TASKA.

Recombinant TASKA was purified to homogeneity using an Ni-NTA affinity chromatography column. The purified TASKA protein exhibited a molecular mass of 50 kDa on sodium dodecyl sulfate-polyacrylamide gel electrophoresis (SDS-PAGE) (Figure 1A). A clear band of 50 kDa was also observed in the zymogram analysis, which indicated the amylolytic activity of the purified sample (Figure 1B).

### 2.2. Optimization of the Immobilization Conditions

The purified TASKA protein was immobilized on four different supports. Several conditions were then tested to determine the effects of pH, temperature, and the amount of enzyme (mg) added per g support on the immobilization performance of each support; the best conditions are summarized in Table 2.

#### 2.2.1. Optimum pH and Temperature

**Figure 2:**
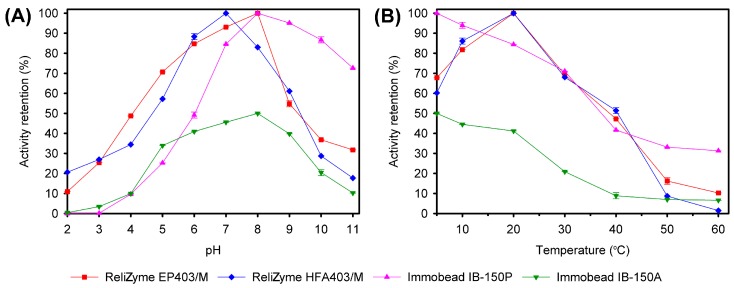
Optimization of TASKA immobilization conditions on 4 different supports. (**A**) Effects of pH on TASKA immobilization; (**B**) Effects of temperature on TASKA immobilization. The values shown represent the mean ± standard error of triplicate analyses.

The TASKA protein bound optimally at pH 8.0 for all the tested supports, except to ReliZyme HFA403/M, which bound TASKA optimally at pH 7.0 (Table 2 and Figure 2A). The optimum immobilization temperatures for the ReliZyme and Immobead supports were 20 °C and 4 °C, respectively (Table 2 and Figure 2B).

#### 2.2.2. Protein-Loading Efficiency

**Figure 3:**
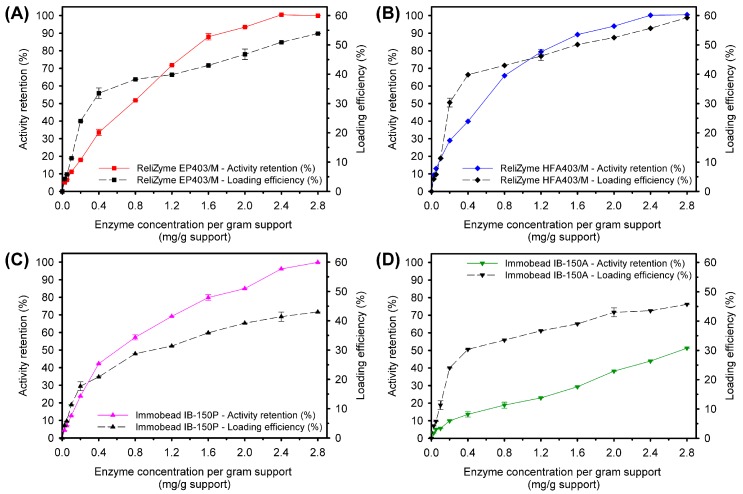
Activity retention and loading efficiency of the immobilized TASKA enzyme on (**A**) ReliZyme EP403/M; (**B**) ReliZyme HFA403/M; (**C**) Immobead IB-150P; and (**D**) Immobead IB-150A at different enzyme concentrations. The values shown represent the mean ± standard error of triplicate analyses.

The protein-loading efficiency experiments showed that the amount of TASKA bound to all tested supports was higher when the offered enzyme concentration was increased (Figure 3). The ReliZyme EP403/M, ReliZyme HFA403/M, Immobead IB-150P, and Immobead IB-150A supports maximally bound 54%, 59%, 43%, and 46% of the offered enzyme (2.8 mg/g support), respectively (see Equation (1) in the Materials and Methods Section), as shown in Table 2 and Figure 3.

#### 2.2.3. Activity Retention and Recovery

Only 51% of the enzyme activity was retained on the Immobead IB-150A beads (see Equation (2) in the Materials and Methods Section, Table 2, and Figure 3). Maximum (100%) activity retention was achieved with ReliZyme EP403/M, ReliZyme HFA403/M, and Immobead IB-150P supports (Table 1). High-activity retention was observed with the ReliZyme EP403/M, ReliZyme HFA403/M, and Immobead IB-150P supports, although the immobilized enzyme on ReliZyme HFA403/M outperformed other supports as its activity recovery was the highest (see Equation (3) in the Materials and Methods Section and Table 2). Enzyme immobilized with ReliZyme EP403/M (46%) and Immobead IB-150P (37%) showed poor activity recoveries, despite the fact that these carriers could hold an equimolar amount of enzyme as could the ReliZyme HFA403/M support (93%) (Table 2 and Figure 3).

### 2.3. Characterization of Free and Immobilized TASKA

#### 2.3.1. Biochemical Characterization

**Figure 4:**
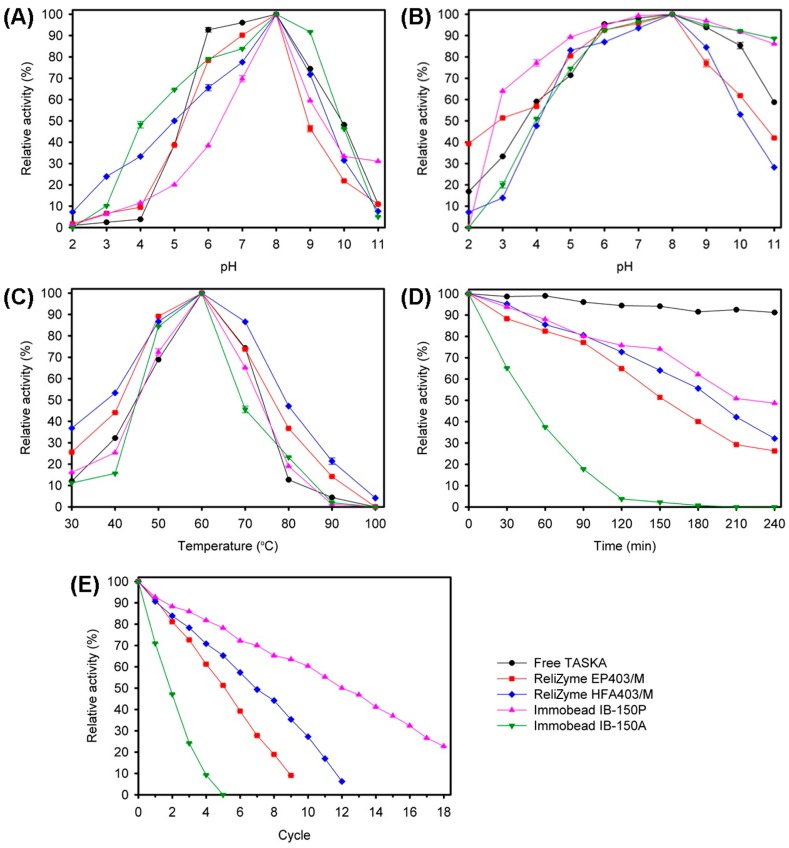
Biochemical characterization of free and immobilized TASKA. Effects of pH on the enzyme (**A**) activity and (**B**) stability; (**C**) Effect of temperature on the enzyme activity; (**D**) Enzyme thermostability at 60 °C; (**E**) Reusability of enzymes at 60 °C. The values shown represent the mean ± standard error of triplicate analyses.

The free and immobilized TASKA enzymes exhibited similar optimum activity at pH 8.0 (Figure 4A). The pH-stability profiles for free and immobilized TASKA varied (Figure 4B). The free enzyme was stable at pH 6.0–9.0. The immobilized TASKA on ReliZyme EP403/M and ReliZyme HFA403/M showed comparable pH stabilities over a pH range of 6.0–8.0. The immobilized enzyme on the Immobead IB-150P and Immobead IB-150A supports exhibited broader pH stabilities of pH 5.0–10.0 and pH 6.0–10.0, respectively.

The free and immobilized TASKA enzymes were optimally active at 60 °C (Figure 4C). The thermostability of free and immobilized TASKA were examined over a 4-h period at 60 °C (Figure 4D). The free enzyme retained 90% of its original activity after the 4-h incubation period. Nevertheless, the thermostability of the immobilized enzyme on all tested supports was lower than that of the free enzyme. The immobilized TASKA enzyme on ReliZyme EP403/M, ReliZyme HFA403/M, Immobead IB-150P, and Immobead IB-150A retained 50% of the initial activity after incubation periods of 150 min, 180 min, 240 min, and 30 min, respectively.

The reusability of immobilized TASKA was studied by measuring the reducing sugars produced from soluble starch hydrolysis at 60 °C (Figure 4E). The immobilized enzyme on ReliZyme EP403/M, ReliZyme HFA403/M, Immobead IB-150P, and Immobead IB-150A retained at least 50% activity after 5, 7, 12, and 1 cycle of reuse, respectively.

#### 2.3.2. Analysis of Reaction Products

**Figure 5:**
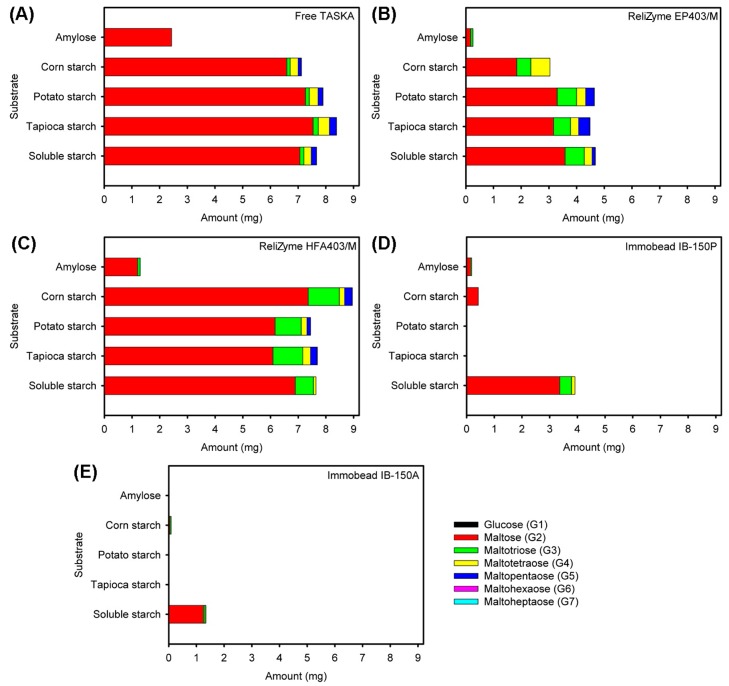
Analysis of reaction products on different substrates by HPLC-ELSD using (**A**) free TASKA and immobilized TASKA on (**B**) ReliZyme EP403/M; (**C**) ReliZyme HFA403/M; (**D**) Immobead IB-150P; and (**E**) Immobead IB-150A.

The free and immobilized TASKA enzymes were reacted with various polysaccharides (i.e., soluble starch, tapioca starch, potato starch, corn starch, and amylose) and the products were analyzed by high performance liquid chromatography with an evaporative light-scattering detector (HPLC-ELSD). In general, maltose (G2) was the major product of all degraded polysaccharides (Figure 5).

With the ReliZyme EP403/M support, the total reducing sugars produced by the immobilized enzyme were reduced by approximately 50%, as compared to the free enzyme (Figure 5A,B). The total reducing sugars produced by the ReliZyme HFA403/M-immobilized enzyme were comparable with the free enzyme (Figure 5A,C), except that 25% more sugars were formed from corn starch. Moreover, the product-specificity spectrum of free TASKA and immobilized enzyme on ReliZyme HFA403/M differed, with a notably higher percentage of maltotriose (G3) produced by the latter.

With Immobead IB-150P and Immobead IB-150A, the immobilized enzymes showed a major change in substrate specificity (Figure 5D,E). The immobilized enzyme on Immobead IB-150P was only able to degrade soluble starch, corn starch, and amylose, forming maltose (G2), maltotriose (G3), and maltotetraose (G4), respectively (Figure 5D). In contrast, the Immobead IB-150A-immobilized enzyme was only able to degrade soluble starch, while a negligible amount of products were detected upon reacting with corn starch (Figure 5E).

## 3. Discussion

### 3.1. Properties of ReliZyme and Immobead Supports

Thermophilic enzymes (thermozymes) exhibit remarkable performance in withstanding elevated temperatures and exhibit resistance to other harsh conditions such as extreme pH, chemical denaturants, and organic solvents [2]. These characteristics are suited to applications in several industries such as the food, biofuel, paper and pulp, and pharmaceutical industries [2,7]. Immobilization of thermozymes may benefit these industries by enhancing enzyme activity and stability (i.e., temperature and pH) as well as promoting enzyme reusability [33]. In addition, the deformation and reduced conformational mobility of the thermozyme structures may alter theirspecificity and enantioselectivity, or in some cases reduce substrate and product inhibition [53].

In the present study, we employed the ReliZyme EP403/M, ReliZyme HFA403/M, Immobead IB-150P, and Immobead IB-150A support systems (Table 1). In general, these carriers are epoxide-activated supports with hydrophilic surfaces, with the exception that the exterior of the Immobead IB-150A support is hydrophobic due to its attached butyl functional groups. The ReliZyme HFA403/M is a heterofunctional-activated hydrophilic carrier that employs amino-epoxide as its functional group. Based on the element radius study by Pyykkö and Atsumi [54], the calculated length for epoxide and amino-epoxide arms are approximately 7.4 Å and 27.8 Å, respectively.

**Figure 6:**
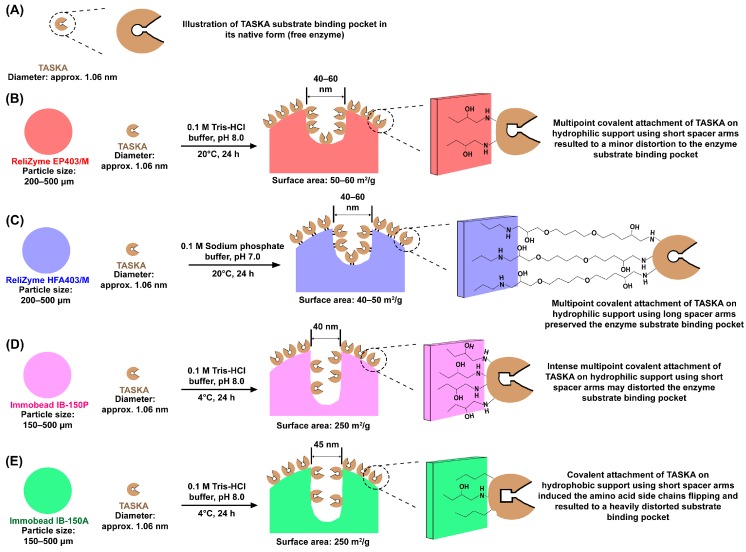
Illustration of (**A**) the substrate-binding pocket of free TASKA and the effect of immobilization on (**B**) ReliZyme EP403/M; (**C**) ReliZyme HFA403/M; (**D**) Immobead IB-150P; and (**E**) Immobead IB-150A towards the immobilized TASKA substrate-binding pocket. The detailed textural properties and functional groups of each support are shown in Table 1. The detailed optimal conditions for TASKA immobilization are shown in Table 2. The diameter of the TASKA enzyme was predicted based on the X-ray crystal structure of the enzyme (PDB ID: 5A2A).

Most supports consist of cylindrical pores. The study conducted by Bayne et al. [55] suggested that supports with pore diameters within 10–100 nm could be categorized into two possible groups, i.e., shallow or deep. As shown in Table 1, all tested supports have pore diameters between 40–60 nm. The surface area of ReliZyme carriers are smaller (40–60 m^2^/g) than those of Immobead carriers (250 m^2^/g) and hence, the pore size of the former may be slightly shallower (Table 1 and Figure 6). Therefore, ReliZyme and Immobead supports have shallow (Figure 6A,B) and deep (Figure 6C,D) pore sizes, respectively. The pore size is regarded as deep for supports with a small surface area or vise verse.

### 3.2. Optimization of Immobilization Conditions

All the tested supports exhibited maximum activity retention (100%), except for the Immobead IB-150A support, which only retained 51% of the enzyme activity (Table 2 and Figure 3). In addition, the activity recovery of the Immobead IB-150A support was the lowest (21%) among the tested supports. This could be related to the hydrophobic surface of Immobead IB-150A (Table 1). TASKA is a soluble enzyme in aqueous solution; thus, the majority of the exterior TASKA structure is hydrophilic [8]. Because the epoxide functional groups (spacer arms) of the carrier are short (7.4 Å) [54], TASKA was bound closely to the Immobead IB-150A surface. It is possible that the different degree of hydrophobicity between the supports and enzymes may have induced amino acid side chain flipping, thus causing a sequential effect on the enzyme conformation and its active site (Figure 6E). This effect has been observed with other enzymes exposed to a hydrophobic interface (such as gas), organic solvents, or high salt concentrations [42,56].

TASKA preferably binds hydrophilic surfaces, yet the activity recoveries of immobilized enzyme on ReliZyme EP403/M, ReliZyme HFA403/M, and Immobead IB-150P varied (Table 2 and Figure 3). This may be due to differences in support pore sizes and functional groups that affected the overall structure of TASKA to different extents, hence causing changes in the biochemical behavior of immobilized TASKA (Figure 6). Collectively from the experimental data, we deduced that the following reasoning could be used to explain these observations.

In general, immobilization on porous materials may improve the operational stability of an enzyme by reducing the exposure to any undesired intermolecular interactions, mechanical stress, or gas/liquid interfaces [20,23,33]. In the case of α-amylase immobilization, several authors have proposed that a matrix with a large pore diameter (70 nm–3μm) [57,58,59,60,61,62] is essential to accommodate the bulky granular size of starches (5–100 nm) [63,64,65]. The pore diameter in this context refers to the opening diameter of the pores. While the pore diameter is definitely an important factor, our experimental data nevertheless suggested that it may not be the sole parameter controlling the performance of immobilized α-amylase.

The immobilized enzyme activity recoveries on ReliZyme EP403/M and ReliZyme HFA403/M (46%–93%) were higher than that of Immobead IB-150P (37%). We inferred that one cause for this difference is differences in the pore size. In this context, the term “pore size” refers to void volume of a pore; nevertheless, no direct measurement of the individual pore sizes are available, but they have been described generally as big or small by Bayne et al. [55]. The pore size is equally important to opening diameter (pore diameter) in various immobilization systems, including the immobilization of TASKA. We deduce that ReliZyme supports may have big pores that are shallow (Figure 6A,B), thus providing better accessibility and higher diffusion freedom for bulky substrates (starch) to react with the bound α-amylase, resulting in greater catalytic efficiency, and this is deduction is supported by earlier reports [35,55,66]. In relative terms, Immobead carriers have smaller, yet deeper pores (Figure 6D,E) than do ReliZyme supports. Enzymes trapped in deeper regions of Immobead supports have reduced interactions with substrates due to poorer mass transfer, which consequently reduces the catalytic efficiency [34,55,58].

The length of the spacer arms of each carrier may also influence the immobilized TASKA activity retention. All of the selected supports have various spacer arm lengths, where the epoxide moiety (approximately 7.4 Å) is shorter than the amino-epoxide moiety (approximately 27.8 Å) (Figure 6) [54]. Poor activity recovery (37%–46% of the original enzyme activity) for immobilized enzymes on ReliZyme EP403/M and Immobead IB-150P supports may relate to the short spacer arms that causes steric hindrance or global structural changes that disfavor a proper conformation of the enzyme substrate-binding pocket, which is adjacent to the active site (Figure 6B,D). In carriers with long spacer arms (i.e., ReliZyme HFA403/M), the multipoint covalent binding of TASKA to the support did not create a major disturbance to the flexibility of the enzyme and; thus, 93% of the original enzyme activity was retained (Figure 6B). This explanation is in agreement with earlier findings on the immobilization of other enzymes, using epoxide- or amino-epoxide-activated supports [20,22,36,38,42].

### 3.3. Characterization of Free and Immobilized TASKA

Immobilized TASKA on all tested supports exhibited relatively similar characteristics (i.e., optimum pH, pH stability, and optimum temperature) as compared to the free enzyme (Figure 4). Similar observations were reported in other enzyme-immobilization studies, the results of which indicated that the immobilized enzymes maintained their free enzyme characteristics [34,36,38,39,41]. The reaction-product analysis indicated that only the immobilized enzyme on ReliZyme HFA403/M produced an amount of reducing sugars from starches that was comparable to the free enzyme (Figure 5).

## 4. Materials and Methods

### 4.1. Materials

All chemicals were of analytical and molecular grade, and were purchased from Merck Millipore (Darmstadt, Germany), unless otherwise stated. Soluble starch was procured from the Kanto Chemical Co. Inc. (Tokyo, Japan). Amylose from potato was purchased from Sigma-Aldrich (St. Louis, MO, USA). ReliZyme EP403/M and ReliZyme HFA403/M were purchased from Resindion S.R.L., Mitsubishi Chemical Corporation (Milan, Italy). Immobead IB-150P and Immobead IB-150A were obtained from ChiralVision (Leiden, The Netherlands).

### 4.2. Expression of TASKA

The *taska* gene was constructed and cloned into pET-28a(+) (Novagen Merck Millipore) using the *Eco*RI and *Xho*I restriction sites, as described by Chai et al. [8]. The pET-28a(+) constructs were transformed into *Escherichia coli* BL21 (DE3) cells (New England BioLabs, Hertfordshire, UK).

To express TASKA, an inoculum was prepared by culturing transformed *E. coli* BL21 (DE3) in Luria-Bertani (LB) medium supplemented with 50 µg/mL kanamycin at 37 °C in an orbital shaker at 200 rpm for 18 h. A 1% (*v*/*v*) inoculum was then transferred into fresh medium and incubated at 37 °C, 200 rpm until the optical intensity at 600 nm reached 0.5, as measured using a 7300 Vis spectrophotometer (Jenway, Staffordshire, UK). Subsequently, enzyme expression was induced by adding a final concentration of 0.4 mM isopropyl-β-D-thiogalactopyranoside, and the culture was further incubated at 37 °C, 200 rpm for 3 h. The culture was then centrifuged (5000× *g*, 10 min, 4 °C) and the cell pellet was lysed using the B-PER™ Bacterial Protein Extraction Reagent Kit (Thermo Fisher Scientific, Rockford, IL, USA). Subsequently, the cell-free lysate was dialyzed against 20 mM sodium phosphate buffer, pH 7.4, for 18 h at 4 °C using SnakeSkin dialysis tubing with a 10-kDa molecular weight cut-off (MWCO; Thermo Fisher Scientific).

### 4.3. Purification of TASKA

TASKA was purified using a pre-packed, 1-mL nickel-nitrilotriacetic (Ni-NTA) Superflow cartridge (Qiagen, Hilden, Germany) equilibrated with 20 mM sodium phosphate buffer, 500 mM NaCl, and 60 mM imidazole (pH 7.4). The bound enzyme was eluted with a linear gradient of 60–500 mM imidazole at a flow rate of 1.0 mL/min. The active fractions were pooled and dialyzed against 100 mM Tris-HCl buffer (pH 8.0) for 18 h at 4 °C.

The molecular mass and purity of TASKA was determined by 12% (*w*/*v*) SDS-PAGE analysis. Zymogram staining for detection of TASKA amylolytic activity was conducted following the method of Yang et al. [67], except that the 1.0% (*w*/*v*) starch solution was prepared in 100 mM Tris-HCl buffer (pH 8.0) and then incubated at 60 °C for 30 min.

### 4.4. Enzyme Activity and Protein-Concentration Assays

α-Amylase activity was determined using the 3,5-dinitrosalicylic acid (DNS) method as described by Miller et al. [68]. With the free enzyme, a reaction mixture consisting of 0.1 mL of enzyme (0.1 U) and 1.0 mL of 1.0% (*w*/*v*) soluble starch in 100 mM Tris-HCl buffer (pH 8.0) was incubated at 60 °C for 10 min. The reaction mixture was quenched on ice for 1 min to stop the reaction. The DNS reagent (1.0 mL) was then added to the mixture. Then, the mixture was boiled for 5 min, and the absorbance at 540 nm was measured. As a control, the absorbance of a reaction mixture lacking the enzyme was measured under the same conditions. With the immobilized enzyme, the enzymatic reaction was done by incubating 0.1 g of immobilized enzyme (0.1 U) with 1.0 mL of 1.0% (*w*/*v*) soluble starch in 100 mM Tris-HCl buffer (pH 8.0) at 60 °C for 10 min. The reaction mixture was quenched on ice for 1 min to stop the reaction. The reaction mixture was then withdrawn and diluted appropriately. Then, 1.0 mL of DNS reagent was added to 1.1 mL of the diluted reaction mixture, boiled for 5 min, and the absorbance was measured at 540 nm. As a control, the wet immobilization support lacking the enzyme was measured under the aforementioned conditions. One unit (U) of α-amylase activity was defined as the amount of enzyme that generated 1 μmol of reducing sugar per min per mL at 60 °C. Maltose was used as the assay standard. Protein concentrations were quantified using a Bicinchoninic Acid (BCA) Protein Assay Kit (Pierce™, Thermo Fisher Scientific) with bovine serum albumin (BSA) as the standard. The enzyme-activity and protein-concentration assays were performed at least in triplicate, unless otherwise specified.

### 4.5. TASKA Immobilization on Different Supports

#### 4.5.1. Immobilization Method

Prior to the immobilization process, the purified TASKA protein was concentrated using the Amicon^®^ Ultra-15 (10 kDa MWCO) centrifugal filter devices (Merck Millipore). The concentrated TASKA protein was then immobilized on 4 different supports (ReliZyme EP403/M, ReliZyme HFA403/M, Immobead IB-150P, and Immobead IB-150A) via covalent attachment. The immobilization process was done by incubating 2.0 g of the respective supports with 5.0 mL of the concentrated TASKA for 24 h under the conditions listed in Table 2. Then, the immobilized TASKA was washed 5 times with the indicated buffer (Table 2) containing a final concentration of 500 mM NaCl to remove the unbound enzymes from the supports. To block the remaining functional groups on the supports, the immobilized enzyme was incubated with 5.0 mL of 3 M glycine under the indicated conditions (Table 2) for 16 h, as described by Mateo et al. [42]. The immobilized TASKA protein was then washed extensively with distilled water to remove traces of glycine. The immobilized TASKA was stored in 100 mM Tris-HCl buffer (pH 8.0) at 4 °C prior to use. The remaining enzyme supernatants and washing solutions were collected and subjected to enzyme-activity and protein-concentration assays. The immobilized TASKA proteins were analyzed for enzyme activity.

#### 4.5.2. Optimization of Immobilization Conditions

##### Optimum pH and Temperature

The optimum pH for immobilization was determined by incubating the enzyme with different supports for 24 h at the indicated temperature (Table 2), using the following buffer (100 mM each): glycine-HCl (pH 2.0–3.0), sodium acetate (pH 4.0–5.5), sodium phosphate (pH 6.0–7.5), Tris-HCl (pH 8.0–9.0), and glycine-NaOH (pH 10.0–11.0). The optimum temperature for immobilization was evaluated at 4–60°C using the respective optimum pH (Table 2) for 24 h.

##### Loading Efficiency

The loading efficiency was determined by incubating TASKA and the respective supports (containing 0.025–2.8 mg of protein per g support) at their respective optimum pH and temperature (Table 2) for 24 h. The loading efficiency was then calculated using the following equation [69]:

Loading efficiency (%) = [(*C*_0_*V*_0_ − *C*_i_*V*_i_ − *C*_j_*V*_j_)/*C*_0_*V*_0_] × 100 (1)

where *C*_0_ is the protein concentration and *V*_0_ is the volume of the free enzyme added to the immobilization process. *C*_i_ is the protein concentration and *V*_i_ is the volume of the remaining enzyme supernatant after immobilization.

##### Activity Retention and Recovery

The optimum activity retention and activity recovery were determined by incubating TASKA with the indicated supports (containing 0.025–2.8 mg of protein per g support) at their respective optimum pH and temperature (Table 2) for 24 h. The activity retention and activity recovery after immobilization were then calculated using Equations (2) and (3), respectively [34,69]:

Activity retention (%) = [*D*/(*A* − *B* − *C*)] × 100 (2)

Activity recovery (%) = (*D*/*A*) × 100 (3)

where *A* is the initial total activity prior to the immobilization process, *B* is the total activity in the remaining supernatant after immobilization, *C* is the total activity in the washing solutions, and *D* is the total activity of the immobilized enzyme.

### 4.6. Characterization of Free and Immobilized TASKA

#### 4.6.1. Biochemical Characterization

The optimum pH of free and immobilized TASKA were determined by incubating the samples with soluble starch dissolved in the following buffers (100 mM each): glycine-HCl (pH 2.0–3.0), sodium acetate (pH 4.0–5.5), sodium phosphate (pH 6.0–7.5), Tris-HCl (pH 8.0–9.0), and glycine-NaOH (pH 10.0–11.0). To determine the pH stabilities, the free and immobilized TASKA were incubated in each buffer without substrate at 25 °C for 30 min, and the residual activities were subsequently measured.

The optimum temperatures of free and immobilized TASKA were evaluated at 30–100 °C at the optimal pH for the enzyme (pH 8.0). The thermostability of free and immobilized TASKA were studied by pre-incubating the samples for 240 min at 60 °C, taking samples at periodic time intervals, and measuring the residual activity under standard assay conditions.

The stability of immobilized TASKA on repeated use was examined by measuring the enzymatic activity on soluble starch at 60 °C. After each enzymatic reaction, the immobilized enzyme was separated and the reaction mixture was subjected to residual activity determination under standard assay conditions. Then, the recovered immobilized enzyme was washed 5 times with 100 mM Tris-HCl (pH 8.0) and stored at 4 °C for 30 min prior to use in another cycle.

#### 4.6.2. Analysis of Reaction Products

The hydrolytic abilities of free and immobilized TASKA were studied by incubating the samples (0.1 U each) for 24 h with soluble starch, tapioca starch, potato starch, corn starch, and amylose (1% *w*/*v* each) in 100 mM Tris-HCl (pH 8.0) using a shaking water bath set at 60 °C and 160 strokes per min. The enzymatic reactions were then stopped by boiling for 10 min. The insoluble particles were filtered through a 0.45-μm nylon membrane syringe filter (Millex-GN, Merck Millipore) and subjected to HPLC-ELSD analysis. Glucose (G1), maltose (G2), maltotriose (G3), maltotetraose (G4), maltopentaose (G5), maltohexaose (G6), and maltoheptaose (G7) (Sigma-Aldrich, St. Louis, MO, USA) were used as standards in the analyses. Non-reacted substrates were used as controls.

The reaction products were analyzed using an Agilent 1260 Infinity HPLC system with an Agilent 1260 Infinity ELSD (Agilent Technologies, Santa Clara, CA, USA), using a 0.5-μm Zorbax carbohydrate analysis (NH_2_) column, 4.6 × 250 mm (Agilent Technologies). The column temperature was maintained at 30 °C. The ELSD nebulizer and evaporator temperatures were maintained at 30 °C, and an N_2_ gas flow of 1.6 standard liters per min (SLM) was used. Acetonitrile:water (65:35, *v*/*v*) was used as the mobile phase at a flow rate of 1.0 mL/min.

### 4.7. Statistical Analysis

The results were analyzed using SYSTAT 12 software (Systat Software Inc., San Jose, CA, USA). Student’s *t*-test yielded a probability value (*p*-value) of less than 0.05, confirming that the data were adequate to test all of the hypotheses.

## 5. Conclusions

Immobilization of α-amylases often relates solely to the support pore diameter used to increase the enzyme immobilization performance. Previously, carriers with large pore diameters (70 nm–3 μm) were proposed to be ideal in accommodating the bulky granular size of starches (5–100 nm) [57,58,59,60,61,62,63,64,65]. This study employed TASKA immobilization on 4 epoxide- or amino-epoxide-activated supports (pores diameter: 40–60 nm), including ReliZyme EP403/M, ReliZyme HFA403/M, Immobead IB-150P, and Immobead IB-150A. Our experimental data indicated that high enzyme activity retention (100%) and activity recovery (93%) could be achieved using ReliZyme HFA403/M. We deduced that this outcome may be related to multiple physical properties of the support (not only the pore diameter), such as its hydrophilic surface, pore size, and extended length (27.8 Å) of its spacer arms (functional groups).

## Figures and Tables

**Table 1:** Main properties of the different supports used in this study ^a^.

Support	Matrix	Surface Properties	Functional Group	Content of Functional Groups (μmol/g)	Particle Size (μm)	Surface Area (m^2^/g)	Density of Functional Groups per Surface Area (μmol/m^2^)	Average Pore Diameter (nm)	Total Pore Volume (mL/g)	Water Retention (%)
ReliZyme EP403/M	Polymethacrylate	Hydrophilic	Epoxide	>30	200–500	50–60	>0.5–0.6	40–60	0.9–1.0	65–75
ReliZyme HFA403/M	Polymethacrylate	Hydrophilic	Amino-epoxide	>40	200–500	40–50	>0.8–1.0	40–60	1.2–1.3	60–70
Immobead IB-150P	Polyacrylic	Hydrophilic	Epoxide	1000	150–500	250	4.0	40	3.0	75
Immobead IB-150A	Polyacrylic	Hydrophobic ^b^	Epoxide	50	150–500	250	0.2	45	3.0	75

^a^ The listed properties were provided by the respective supplier. ^b^ The support contains butyl functional group, which provides hydrophobicity to the support surface.

**Table 2:** Immobilization efficiency of TASKA on different supports.

Support	Optimum Immobilization Condition ^a^					Immobilization Performance		
	Buffer Type (100 mM)	pH	Temperature (°C)	Offered Protein Concentration per gram Dry Support (mg/g Dry Support)	Offered Enzyme Activity per gram Dry Support (U/g Dry Support) ^b^	Protein Loading Efficiency (%) ^c^	Activity Retention (%) ^d^	Activity Recovery (%) ^e^
ReliZyme EP403/M	Tris-HCl	8.0	20	2.8	353	54	100	46
ReliZyme HFA403/M	Sodium phosphate	7.0	20	2.8	353	59	100	93
Immobead IB-150P	Tris-HCl	8.0	4	2.8	353	43	100	37
Immobead IB-150A	Tris-HCl	8.0	4	2.8	353	46	51	21

^a^ The parameters shown correspond to the optimal binding conditions of the TASKA enzyme on the respective supports (see also Figure 2 and Figure 3). ^b^ The offered enzyme activity corresponds to the offered protein concentration. ^c^ Optimum protein-loading efficiency after immobilization on the respective supports (see also Figure 3). ^d^ Optimum activity retention after immobilization on the respective supports (see also Figure 3). ^e^ Relative activity of the immobilized enzyme with respect to the initial activity of the offered enzyme, prior to the immobilization process.

## Abbreviations

The following abbreviations are used in this manuscript:

ASKA: α-Amylase from *Anoxybacillus* sp. SK3-4
BCA: Bicinchoninic acid
BSA: Bovine serum albumin
DNS: 3,5-Dinitrosalicylic acid
G1: Glucose
G2: Maltose
G3: Maltotriose
G4: Maltotetraose
G5: Maltopentaose
G6: Maltohexaose
G7: Maltoheptaose
GH13: Glycoside hydrolase 13
HPLC-ELSD: High performance liquid chromatography with an evaporative light scattering detector
LB: Luria-Bertani
MWCO: Molecular weight cut-off
Ni-NTA: Nickel-nitrilotriacetic
SDS-PAGE: Sodium dodecyl sulfate-polyacrylamide gel electrophoresis
SLM: Standard liters per min
TASKA: Truncated α-amylase from *Anoxybacillus* sp. SK3-4
